# Attenuation of renal ischemic reperfusion injury by salvianolic acid B via suppressing oxidative stress and inflammation through PI3K/Akt signaling pathway

**DOI:** 10.1590/1414-431X20175954

**Published:** 2017-05-15

**Authors:** Z.G. Ma, H.Q. Xia, S.L. Cui, J. Yu

**Affiliations:** 1 Department of Critical Care Medicine, Laiwu Steel Group Hospital, Laiwu City, Shandong, China; 2 Department of Renal Rheumatology, Laiwu Steel Group Hospital, Laiwu City, Shandong, China; 3 Department of Internal Medicine, Laiwu Steel Group Hospital, Laiwu City, Shandong, China

**Keywords:** Renal ischemic reperfusion, Salvianolic acid B, Oxidative stress, Inflammation, PI3K/Akt pathway

## Abstract

Salvianolic acid B (SAB) is one the major phytocomponents of Radix *Salvia miltiorrhiza* and exhibit numerous health promoting properties. The objective of the current study was to examine whether SAB exerts a renoprotective effect by attenuating oxidative stress and inflammatory response through activating phosphatidylinositol 3-kinase/serine-threonine kinase B (PI3K/Akt) signaling pathway in a renal ischemic reperfusion rat model. Forty Sprague-Dawley male rats (250–300 g) were obtained and split into four groups with ten rats in each group. The right kidney of all rats was removed (nephrectomy). The rats of the Control group received only saline (occlusion) and served as a sham control group, whereas rats subjected to ischemic reperfusion (IR) insult by clamping the left renal artery served as a postitive control group. The other 2 groups of rats were pretreated with SAB (20 and 40 mg·kg^-1^·day^-1^) for 7 days prior IR induction and served as treatment groups (SAB 20+IR; SAB 40+IR). Renal markers creatinine (Cr) and blood urea nitrogen (BUN) were significantly lower in the groups that received SAB. Pretreatment with SAB appears to attenuate oxidative stress by suppressing the production of lipid peroxidation products like malondialdehyde as well as elevating antioxidant activity. The concentration of inflammatory markers and neutrophil infiltration (myeloperoxidase) were significantly decreased. Meanwhile, PI3K protein expression and pAkt/Akt ratio were significantly upregulated upon supplementation with SAB, indicating its renoprotective activity. Taken together, these results indicate that SAB can therapeutically alleviate oxidative stress and inflammatory process via modulating PI3K/Akt signaling pathway and probably ameliorate renal function and thus act as a renoprotective agent.

## Introduction

Renal ischemia-reperfusion injury (RIRI) is the major contributor to acute kidney injury (AKI) in different clinical conditions, especially post-renal transplantation, vascular surgeries, trauma and partial nephrectomy (1). Epidemiological studies also indicate that AKI is strongly affiliated with high mortality and morbidity (2). Pathophysiology of RIRI and subsequent AKI is not completely explored till today. However, experimental data suggest that hypoxia, apoptosis, endothelial dysfunction, oxidative stress and inflammatory response play a pivotal role in renal dysfunction during ischemic reperfusion (IR) condition (3,4). Nevertheless, oxidative stress and inflammation are interlinked and thus demonstrate to be the major contributors to RIRI (5,6). The quest for a novel pharmaceutical drug for treating RIRI is increasing enormously due to its strong correlation with AKI.

Radix *S. miltiorrhiza* is a perennial herb, and its dried roots (Danshen) are commonly used in traditional Chinese medicine to treat various cerebrovascular and cardiovascular diseases as well as chronic renal failure (fibrosis) and dermal disorders (7,8). Salvianolic acid B (SAB; CAS:121521-90-2) is one of the abundant active components from water soluble Radix *Salvia miltiorrhiza.* SAB has been reported to show several beneficial properties like antioxidant, anti-inflammatory, anti-apoptotic and anticancer activities (9,10). Previous experiments have indicated that SAB can act as a cardioprotective, hepatoprotective, and neuroprotective agent in the IR model through suppressing oxidative stress, inflammation, and apoptosis (11–13). Moreover, SAB is highlighted to repair the renal tubular epithelial cell in the fibrotic cell model (14). Recently, SAB has been reported to exert its renoprotective activity against iodinated contrast media-induced renal injury rat model through its antioxidant property, via the PI3K/Akt/Nrf2 pathway (15).

Cell protection or survival rate is highly regulated by phosphatidylinositol-3 kinase/serine-threonine kinase B (PI3K/Akt) signal pathway via altering several downstream molecules (16). Numerous scientific studies also inferred that many pharmaceutical or nutraceutical products with potent antioxidant and anti-inflammatory activity can positively regulate various signaling molecules of PI3K/Akt pathway supporting its renoprotective activity (17–19). Thus, we hypothesized that SAB can exhibit its renoprotection via its antioxidant and anti-inflammatory activity mediated by the PI3K/Akt signaling pathway. The present experiment aimed to determine the antioxidant and anti-inflammatory efficacy of SAB through determining antioxidant status, inflammatory markers, renal markers, protein expression of PI3K and Akt as well as renal histological analysis in the RIRI rat model.

## Material and Methods

### Chemicals

Sodium dodecyl sulfate (SDS), tetramethylethylenediamine (TEMED), SAB, formalin, hematoxylin and eosin (H&E) stain were purchased from Sigma-Aldrich (USA). Bromophenol blue, glycerol, ketamine, pentobarbital sodium and hydrogen peroxide were procured from Kangchen Biotechnology (China). All the other chemicals used were of analytical grade.

### Experimental animals

Forty healthy Sprague-Dawley male adult rats weighing 250–300 g were maintained in the animal house of Laiwu Steel Group Medical Hospital. Rats were housed in a steel cage under a 12/12 h light/dark cycle with free access to food and water. All experimental procedures were approved by the ethical committee of Laiwu Steel Group Hospital (LSGHU672) and carried out in accordance with National Institutes of Health Guidelines.

### IR insult/induction

After 2 weeks of assimilation and an overnight fast, rats were anesthetized with ketamine (50 mg/kg) and pentobarbital sodium (20 mg/kg) intraperitoneally (*ip*) before surgery. All rats underwent right nephrectomy. Rats were placed on a warm pad, and the body temperature was maintained at 37°C throughout the surgical procedure. Then the hair near the abdomen was shaved and the skin disinfected with 70% ethanol, and the surgical procedure was carried out in a sterile condition. A bilateral flank incision was made gently exposing both kidneys and the right kidney was removed (nephrectomy). The left renal artery (pedicle) was occluded by clamping for 50 min to induce ischemia followed by reperfusion for 48 h by removing the clamp to restore the blood flow. The wound was closed by 4-0 nylon suture and the animals were transferred to the respective cage and allowed to recover. Sham-controlled rats were not occluded or clamped. The average systolic and diastolic blood pressure and heart beat were continuously monitored to confirm the health status.

### Animal grouping

Group I: rats receiving saline (sham-operated control) without clamping or occlusion; group II: rats underwent ischemic reprerfusion (IR) insult; groups III and IV: rats were pretreaded with SAB (20 and 40 mg·kg^-1^·day^-1^, respectively) by dissolving it in saline and *ip* injection for 6 days and 1 h prior to IR insult (on the 7th day) and thus served as treatment groups (SAB 20+IR; SAB 40+IR).

### Sample preparation

On the 3rd day after IR insult, rats were sacrificed by cervical decapitation under pentobarbital sodium (40 mg/kg) anesthesia (*ip*) and blood samples were collected for evaluating various biochemical parameters. Renal tissues were harvested immediately and washed with ice-cold saline; a portion of tissue was fixed in 10% formalin for histological analysis and the remaining portion was homogenized (10%) using phosphate buffered saline (PBS) and used for biochemical analysis.

### Biochemical analysis

#### Renal function test

Serum creatinine (Cr) and blood urea nitrogen (BUN) levels were determined by a commercial assay kit from Nanjing Jiancheng Bioengineering Institute (China) following the manufacturer's protocol.

#### Antioxidant enzymes and lipid peroxidation products

The activities of superoxide dismutase (SOD), glutathione contents (GSH) and catalase (CAT) were assayed in renal tissues (homogenate) using commercial kits based on the supplier instructions (Nanjing Jiancheng Bioengineering Institute). Similarly, lipid peroxidation products like malondialdehyde (MDA) levels were also evaluated in renal tissue using a commercial kit provided by Shanghai Yantuo Biotechnology Ltd.

#### Myeloperoxidase (neutrophil infiltration) and other inflammatory markers

Myeloperoxidase (MPO) activity in renal tissue was measured using an MPO assay kit (Nanjing Jiancheng Bioengineering Institute) based on manufacturer's procedure. One unit of MPO activity was defined as the amount of enzyme degrading 1 mmol peroxidase/min at 25°C and is reported as unit per gram (U/g) of wet tissue. The levels of NF-κB free p65 subunit (NF-p65) in the nuclear fraction (Cell Biolabs Nuclear/Cytosolic Fractionation Kit, USA) of the renal tissue were measured by ActivELISA kit from Imgenex (Novus Biologicals, USA). The concentrations of proinflammatory cytokines IL-1β, IL-6 and TNF-α in renal tissue were quantified using commercial ELISA kits based on the manufacturer's protocols (Thermo Fisher Scientific Inc., USA).

### Western blotting

Western blot techniques were employed to assess the protein expressions of PI3K, Akt and pAkt in renal tissue (homogenates). Protein levels were estimated by BCA protein assay kit from Biovision (USA). A 50-µg aliquot of protein was loaded equally in each well on 10% polyacrylamide gels and electrotransferred to polyvinylidene difluoride membrane (Amersham Pharmacia Biotech, USA) at room temperature by blocking with a tris-buffered saline (TBS) solution, which contains tween 20 and 5% skimmed milk. The membrane was incubated at 4°C for 2 h with different primary antibodies like rabbit polyclonal anti-PI3K (1:1000; Santa Cruz Biotechnology, USA), anti-Akt and pAkt antibody (Ser 473-1:2000; Cell Signaling Technology, USA) or rabbit monoclonal anti-rat β-actin (1:500; Zhongshan Biotechnology, China), which served as internal control, and washed with TBS. A secondary antibody was conjugated with horseradish peroxidase-linked anti-rabbit antibody (1:2000 Santa Cruz Biotechnology) in TBS at room temperature for 1 h, and washed twice with TBS to remove unbound antibodies. The bounded antibodies were visualized using an enhanced chemiluminescence western blotting detection kit (INtRON Biotechnology Co., Ltd., Korea) and the protein expression (band) was quantified using the Image-Pro Plus software (Media Cybernetics, Inc., USA).

### Histomorphological evaluation

A section of renal tissue sample was removed immediately from each experimental rat and fixed in 10% buffered formalin for 24 h, and then sequentially dehydrated using descending grades of isopropanol and xylene, and embedded in paraffin wax. A 5-μm thick slice of embedded renal tissues was made using a microtome, mounted onto a microscopic slide and stained with H&E. The renal tissue slides were then examined under a light microscope (Leica DM 6000B, Leica Microsystems, Germany) to evaluate histomorphological changes by a pathologist blind to the experimental groups. The histological changes (renal damages) were evaluated based on percentage of tubular damage (tubular necrosis, epithelial denudation and swelling) as 0% (normal tubular morphology), 25% (minimal tubular damage/swelling), 50% (mild tubular damage/swelling), 75% (moderate tubular damage/swelling), and 100% (severe tubular damage/swelling) according to the method of Hu et al. (20).

### Statistical analysis

Results are reported as means±SD for 10 rats in each group. Variation between groups was evaluated by one-way analysis of variance (ANOVA) and least significant difference (LSD) was measured using *post hoc* multiple comparison tests. The Statistical Package for the Social Sciences (SPSS; 23) provided by IBM (USA) was used and a P value less than 0.05 was considered to be statistically significant.

## Results

### Renal markers

A pronounced increase (P<0.01) in the levels of Cr and BUN was noted in the IR group compared to sham control rats. However, pretreated SAB (20 and 40 mg/kg) rats presented a significant decrease (P<0.01) in those renal markers, thus showing its renoprotective action (Table 1).

**Table 1.** Effect of salvianolic acid B (SAB) on the levels of renal markers in experimental groups.

Group	Creatinine (mg/dL)	BUN (mg/dL)
Sham control	0.69 ± 0.07	18.44 ± 2.10
IR	1.32 ± 0.18^a^ **	29.49 ± 3.20^a^ **
SAB 20 + IR	0.92 ± 0.11^b^ **	24.53 ± 3.06^b^ **
SAB 40 + IR	0.84 ± 0.08^b^ **	23.12 ± 2.80^b^ **

Data are reported as means±SD for 10 rats in each group.

** P<0.01:

a IR group compared to the sham control group;

b treatment groups (SAB 20, 40 mg·kg^-1^·day^-1^) compared to the IR insulted group. SAB: Salvianolic acid B; BUN: blood urea nitrogen; IR: ischemic reperfusion.

### Renal antioxidants and lipid peroxidation products

The renal SOD, GSH, and CAT activities were significantly reduced in the IR group (P<0.01), whereas MDA levels were significantly enhanced (P<0.01) similar to the sham control group. Both SAB 20 and 40 groups had elevated activities of SOD, GSH and CAT as well as suppressed MDA levels (P<0.05). However, SAB 40 group showed better (P<0.05) antioxidant capacity than SAB 20 (Table 2).

**Table 2.** Effect of salvianolic acid B (SAB) on the activities of renal antioxidants and lipid peroxidation products in experimental groups.

Group	SOD (U/mg ptn)	CAT (U/mg ptn)	GSH (µg/mg ptn)	MDA (nmols/mg ptn)
Sham control	4.02 ± 0.54	62.46 ± 8.10	10.02 ± 1.21	0.79 ± 0.12
IR	2.48 ± 0.35^a^ **	50.35 ± 6.64^a^ **	7.11 ± 0.82^a^ **	1.34 ± 0.10^a^ **
SAB 20 + IR	3.25 ± 0.24^b^ **	57.45 ± 5.06^b^ *	9.08 ± 0.63^b^ *	0.95 ± 0.09^b^ *
SAB 40 + IR	3.68 ± 0.97^b^ **	60.83 ± 9.45^b^ ** c *	9.36 ± 0.88^b^ *	0.82 ± 0.07^b^ ** c *

Data are reported as means±SD for 10 rats in each group.

** P<0.01,

* P<0.05

a IR group compared to the sham control group;

b treatment groups (SAB 20 and 40 mg·kg^−1^·day^−1^) compared to the IR insulted group;

c SAB 40 group compared to the SAB 20 group. One unit (U) of SOD activity was defined as the amount of enzyme required to inhibit 50% dismutation of the superoxide radical at 550 nm. One unit (U) of CAT activity was defined as the amount of enzyme required to quench 50% of H_2_O_2_ radicals at 405 nm. ptn: protein; IR: ischemic reperfusion; SOD: superoxide dismutase; GSH: glutathione contents; CAT: catalase; MDA: malondialdehyde.

### Renal MPO

**Figure 1.**
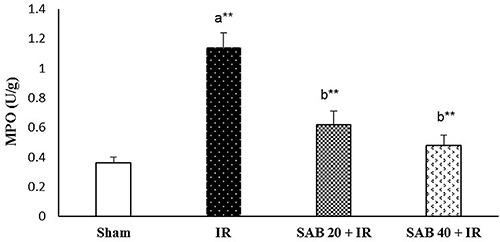
Effect of salvianolic acid B (SAB) on renal myeloperoxidase (MPO) levels in experimental groups. Data are reported as means±SD for 10 rats in each group. **P<0.01. *a*, ischemia-reperfusion (IR) group compared to the sham control group; *b*, treatment groups (SAB 20 and 40 mg·kg^-1^·day^-1^) compared to the IR group (ANOVA).

Figure 1 depicts the effect of SAB on renal MPO levels in experimental rats. The rats in the IR group had increased (P<0.01) levels of MPO compared to sham control rats. Both SAB 20 and 40 groups had decreased MPO levels, demonstrating its anti-inflammatory activity (P<0.01). There were no significant differences between SAB 40 and SAB 20 groups.

### Renal inflammatory markers

Table 3 illustrates the effect of SAB on the activities of renal inflammatory markers in experimental rats. The concentrations of inflammatory markers nuclear factor NF-p65 subunit of NF-κB, IL-1β, IL-6 and TNF-α in renal tissue of IR-induced rats were markedly increased (P<0.01) compared to sham control rats. In comparison with IR rats, SAB treatment (20 and 40) lowered the concentrations of those inflammatory markers (P<0.01). Nevertheless, SAB 40+IR treatment significantly reduced (P<0.05) the concentrations of those inflammatory markers compared to SAB 20+IR treated rats.

**Table 3.** Effect of salvianolic acid B (SAB) on the activities of renal inflammatory markers in experimental groups.

Group	NF-p65 (pg/mg protein)	IL-1β (ng/mg protein)	IL-6 (pg/mg protein)	TNF-α (ng/mg protein)
Sham control	95.82 ± 9.12	81.55 ± 9.81	91.39 ± 8.67	98.86 ± 10.32
IR	211.35 ± 23.93^a^ **	198.34 ± 16.13^a^ **	232.38 ± 31.24^a^ **	272.13 ± 21.45^a^ **
SAB 20 + IR	125.85 ± 10.01^b^ **	121.83 ± 12.93^b^ **	136.67 ± 12.62^b^ **	167.13 ± 18.23^b^ **
SAB 40 + IR	101.45 ± 12.83^b^ ** c *	96.94 ± 9.56^b^ ** c *	112.43 ± 11.73^b^ ** c *	130.11 ± 12.44^b^ * c *

Data are reported as means±SD for 10 rats in each group.

* P<0.05,

** P<0.01

a IR group compared to the sham control group;

b treatment groups (SAB 20 and 40 mg·kg^−1^·day^−1^) compared to the IR insulted group;

c SAB 40 group compared to the SAB 20 group (ANOVA). IR: ischemic reperfusion; NF-p65: nuclear factor p65 subunit; IL-1β: interleukin 1β; IL-6: interleukin 6; TNF-α: tumor necrotic factor α.

### Protein expression

**Figure 2.**
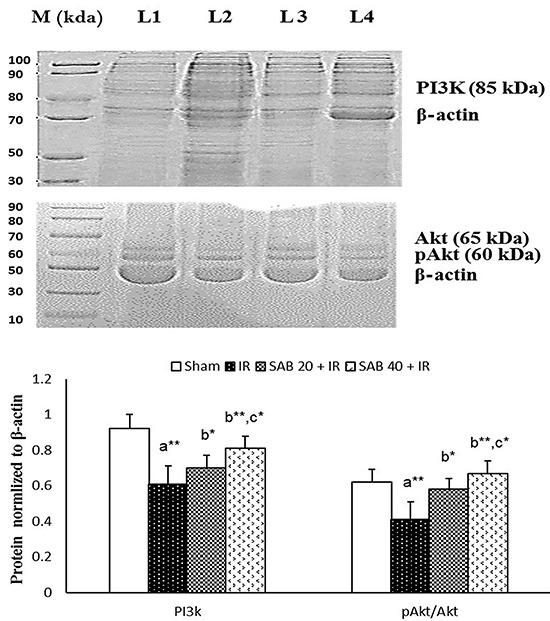
Effects of salvianolic acid B (SAB) on protein expression of PI3K, and pAkt/Akt ratio in renal tissue (homogenate) of experimental groups. Data are reported as means±SD for 10 rats in each group. β-actin was used as the internal standard. M represents molecular ladder/marker ranging from 100 to 10 kDa; L1 represents sham control group; L2 represents ischemia-reperfusion (IR) insulted group; L3 represents SAB 20 mg·kg^-1^·day^-1^group; L4 represents SAB 40 mg·kg^-1^·day^-1^ group. *P<0.05 and **P<0.01: *a*, IR group compared to the sham control group; *b*, treatment groups (SAB 20, 40) compared to the IR insulted group; *c*, SAB 40 group compared to the SAB 20 group (ANOVA).

There was a significant downregulation (P<0.01) of renal PI3K, Akt and pAkt (pAkt/Akt ratio) protein expression in the IR rats compared to the sham group. Pretreatment with SAB (20 and 40) on IR-induced rats caused upregulation (P<0.01) of the levels of PI3K, Akt and pAkt (pAkt/Akt ratio) similar to the IR group. However, SAB 40 significantly upregulated (P<0.05) the protein expression of PI3K, Akt and pAkt (pAkt/Akt ratio) compared to SAB 20 (Figure 2).

### Renal morphology

**Figure 3.**
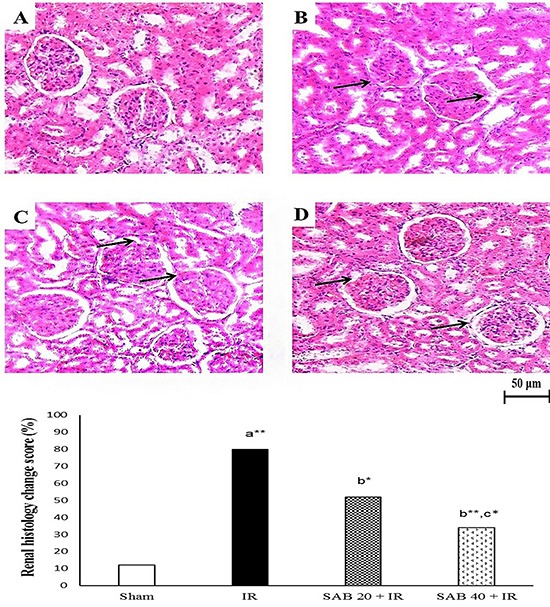
Effect of salvianolic acid B (SAB) on the renal section with hematoxylin and eosin staining in experimental groups (400×) as well as renal histological change score. The transection of renal tissue of sham-controlled rats showed normal glomerulus architecture and distal and proximal convoluted tubules of the nephron (*A*). The transection of ischemia-reperfusion (IR) insulted rats showed the presence of highly swollen renal tubules with epithelial denudation of basement membrane (arrow) and few necrotic tubules (*B*). The transection of SAB 20+IR rats shows moderate renal tubular swallowing and less epithelial denudation (arrow) compared to the IR insulted group (*C*). SAB 40+IR pretreated rats showed better tubular morphology with few swollen tubules and the structure of renal tissue was almost similar to sham control rats (*D*) without any glomerular hypertrophy or necrotic tubules. Data are reported as means±SD. *P<0.05, **P<0.01: *a*, IR group compared to the sham control group; *b*, treatment groups (SAB 20 and 40 mg·kg^-1^·day^-1^) compared to the IR insulted group; *c*, SAB 40 group compared to the SAB 20 group (ANOVA).

The transection of renal tissue of sham control rats showed a normal glomerulus architecture (Figure 3A; 400×). The transection of IR rats (Figure 3B) revealed the presence of highly swollen renal tubules with epithelial denudation of the basement membrane (glomerular hypertrophy) and few necrotic tubules. Thus, the IR group had a higher renal damage score (P<0.01) compared to the sham control group. Transection of SAB 20+IR rats showed moderate renal tubular swelling and less epithelial denudation (Figure 3C). SAB 40+IR rats showed better tubular morphology with mild swollen tubules, and the structure of renal tissue was almost similar to sham control rats without any glomerular hypertrophy or necrotic tubules (Figure 3D). Hence, this indicated that SAB 40 treatment lowered the renal damage score significantly (P<0.05) compared to SAB 20.

Overall, SAB 40-treated rats demonstrated much better renoprotective activity in terms of suppressing oxidative stress and inflammation and thereby improving renal function, which is evidenced from histomorphological evaluation and other biochemical analysis, compared to SAB 20.

## Discussion

**Figure 4.**
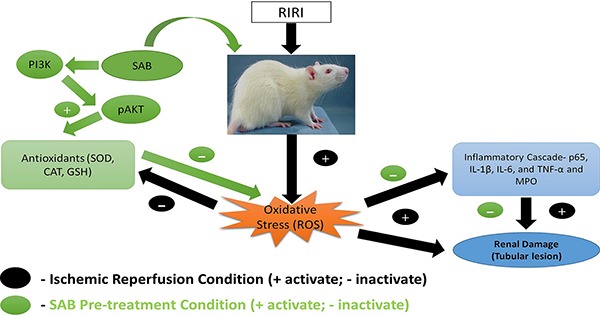
Underlying mechanism for the renoprotective effect of salvianolic acid B (SAB). RIRI: renal ischemic reperfusion injury; PI3K: phosphatidylinositol 3-kinase; pAkt: phosphorylated Akt; SOD: superoxide dismutase; GSH: glutathione contents; CAT: catalase; MPO: myeloperoxidase; p65: nuclear factor p65 subunit; IL-1β: interleukin 1β; IL-6: interleukin 6; TNF-α: tumor necrotic factor α.

Numerous studies have reported that SAB shows positive results against the IR model in different organs (11,13,21). Moreover, several studies confirm that SAB can substantially ameliorate renal function by lowering inflammation and oxidative stress via several signaling pathways (9,15,21). Nevertheless, the exact underlying mechanisms behind the renoprotective effect of SAB in the IR model are still obscure. Few researchers indicated that both SAB and lithospermic acid B (LAB) have a similar structure with different spatial configuration and hence exhibit a similar effect in various animal models (9,10). The underlying mechanism for the renoprotective effect of SAB is represented in Figure 4.

Ischemic insulted rats showed increased levels of Cr and BUN compared to the sham control group. Since the ROS production is exceedingly high during IR, free radicals damage nephron epithelia as well as alter the microcirculatory system (afferent and efferent), and thus reduce the glomerular filtration rate (GFR) (22,23). Animals pretreated with SAB (20 and 40 mg·kg^-1^·day^-1^) showed a considerable decrease in the levels of Cr and BUN, due to its free radical scavenging activity. However, no notable change was observed between the SAB treatment groups. Kang et al. (21) also indicated that LAB could lower renal markers like Cr by suppressing excessive free radicals in an IR rat model and thereby improving Cr clearance rate and GFR. This finding indicated that SAB pretreatment can effectively protect the renal tissue against RIRI.

The activities of SOD, GSH, and CAT in renal tissues of IR rats were significantly decreased. These endogenous antioxidants are highly utilized to counter the excessive free radicals, and hence the activity of these antioxidants are significantly reduced in IR group. SAB (20 and 40) administration greatly enhanced antioxidant activity. However, the SAB 40 group showed significantly greater antioxidant activity than SAB 20 group. Zhao et al. (24) pointed out that salvianolic acid exhibits greater free radical scavenging and antioxidant activity than other major phytocomponents of *S. miltiorrhiza.* In addition, Tongqiang et al. (15) proved that SAB can significantly upregulate Nrf2 (nuclear factor erythroid-2-related factor) protein expression through activating PI3K/Akt pathway and thus enhancing the production of endogenous antioxidants.

MDA is considered to be a lipid peroxidation product and hence used as an indicator for evaluating oxidative stress (12). Our results were similar to the study by Chen and Zhang (25), who indicated that SAB could substantially inhibit lipid peroxidation with enhanced free radical scavenging activity in ischemia-reperfusion model. Moreover, Soung et al. (26) reported that SAB/LAB, with seven free hydroxyl groups and fewer double bonds, would possess better free radical scavenging and anti-nitration activity than other active components of *S. miltiorrhiza.*

Several researchers have pointed out that excessive free radical generation and altered inflammatory response are the hallmarks of RIRI, as they are interlinked with each other (5,27). MPO is a neutrophil-specific enzyme, which would increase during activation of neutrophils (infiltration) and hence, it is used as a surrogate marker for detecting inflammation (28). In our study, the rats in the IR group had increased levels of MPO compared to sham control rats. During renal ischemic condition, neutrophils start to infiltrate into the damaged renal tissue (due to elevated oxidative stress) and stimulate pro-inflammatory markers, which would further promote neutrophil infiltration. This process blocks the microcirculation in the renal tissue, resulting in further renal damage and thereby affecting GFR (22,23). Both SAB 20 and 40 concomitantly reduced the MPO levels to near normal, due to its anti-inflammatory and antioxidant activity. Han et al. (9) also demonstrated that pretreatment with SAB can significantly impede the neutrophil infiltration/activation (MPO) as well as mast cell activation and thus halt the subsequent inflammatory cascade.

The concentrations of inflammatory markers in renal tissues of the IR group were considerably higher due to increasing neutrophil infiltration and oxidative stress (29,30). Both SAB 20 and 40 lowered the inflammatory markers, but SAB 40 treatment showed better anti-inflammatory activity than SAB 20. We speculate that rats pretreated with SAB (20 and 40) might abolish the concentrations of these pro-inflammatory cytokines and NF-p65 by blocking the translocation of p65 (an active nuclear subunit of NF-κB) from the cytosol into the nucleus as well as blocking DNA binding capacity of NF-p65. Thus, SAB inhibited the activation of NF-p65 and thereby downregulated the expression of various pro-inflammatory proteins like TNF-α, IL-1β, and IL-6, confirming its anti-inflammatory property.

Our results are in accordance with Zhang and Wang (31) who reported that SAB can inhibit TNF-α (an inflammatory mediator) via suppressing ROS generation in a cell line model. Furthermore, SAB is reported to inhibit the activation of NF-κB and other inflammatory cytokines in carbon tetrachloride induced hepatic fibrosis rat model (32).

An impressive number of scientific experiments indicated that some pharmaceutical or nutraceutical products with potent antioxidant and anti-inflammatory activity can positively modulate various signaling molecules of PI3K/Akt pathway (17–19). Our finding showed that SAB can activate Akt by phosphorylating this pathway, and hence the pAkt/Akt ratio was significantly elevated, protecting the renal cell from excessive oxidative stress and inflammatory process. Our results are in agreement with Tongqiang et al. (15), who also reported that SAB could display its renoprotective activity through its antioxidant property, which mediated the PI3K/Akt/Nrf2 pathway in iodinated contrast media-induced renal injury rat model. Previous studies have demonstrated that SAB can protect the endothelial cells against hydrogen peroxide toxicity (endogenous ROS) via upregulating PI3K and pAkt signaling molecule (33).

Previously, it has been documented that in IR-insulted condition the levels of inflammatory markers like TNF-α, IL-1β, and IL-6 (cytokines) are substantially elevated, which stimulates the activation of various endothelial adhesion molecules. This disturbs the renal microcirculation and results in damage of renal tubular epithelium (triggering tunular necrosis), eventually causing renal damage or injury (34). SAB 40 treatment significantly lowered the histological renal damage score. Our data are in agreement with Kang et al. (21), who demonstrated that SAB treatment could effectively improve renal function by upregulating Na^2+^/K^+^ ATPases pump and significantly suppress the excessive free radicals via its antioxidant activity. Likewise, SAB/LAB was reported to abolish the progression of glomerular hypertrophy via suppressing ROS production in a diabetic rat model (35).

Since this study concentrated more on oxidative stress and inflammation, mitochondrial and endothelial dysfunction were not assessed, which is a study limitation. Moreover, renal functions like GFR and renal pumps activity were not measured. We have not utilized any standard renoprotective drug to compare the effect of SAB. However, our hypothesis is well supported by our results. The renoprotective effect exerted by SAB (especially at 40 mg·kg^-1^·day^-1^) can be attributed to its antioxidant and anti-inflammatory activity, stimulated by effective activation of PI3K and Akt (pAkt) that results in attenuation of oxidative stress and inflammatory response in a RIRI rat model. Further studies are required to explore the entire molecular mechanism behind the renoprotective activity of SAB.
